# Maximum a posteriori Bayesian methods out-perform non-compartmental analysis for busulfan precision dosing

**DOI:** 10.1007/s10928-024-09915-w

**Published:** 2024-03-23

**Authors:** Jasmine H. Hughes, Janel Long-Boyle, Ron J. Keizer

**Affiliations:** 1 InsightRX, 548 Market St. #88083, San Francisco, CA 94104 USA; 2 Department of Clinical Pharmacy, University of California, San Francisco, CA USA; 3 Department of Pediatrics, Division of Allergy, Immunology, and Bone Marrow Transplantation, University of California, San Francisco, CA USA

**Keywords:** Model-informed precision dosing, Non-compartmental analysis, Pharmacokinetics, Bayesian forecasting

## Abstract

Dose personalization improves patient outcomes for many drugs with a narrow therapeutic index and high inter-individuality variability, including busulfan. Non-compartmental analysis (NCA) and model-based methods like maximum a posteriori Bayesian (MAP) approaches are two methods routinely used for dose optimization. These approaches vary in how they estimate patient-specific pharmacokinetic parameters to inform a dose and the impact of these differences is not well-understood. Using busulfan as an example application and area under the concentration–time curve (AUC) as a target exposure metric, these estimation methods were compared using retrospective patient data (*N* = 246) and simulated precision dosing treatment courses. NCA was performed with or without peak extension, and MAP Bayesian estimation was performed using either the one-compartment Shukla model or the two-compartment McCune model. All methods showed good agreement on real-world data (correlation coefficients of 0.945–0.998) as assessed by Bland–Altman plots, although agreement between NCA and MAP methods was higher during the first dosing interval (0.982–0.994) compared to subsequent dosing intervals (0.918–0.938). In dose adjustment simulations, both NCA and MAP estimated high target attainment (> 98%) although true simulated target attainment was lower for NCA (63–66%) versus MAP (91–93%). The largest differences in AUC estimation were due to different assumptions for the shape of the concentration curve during the infusion phase, followed by how the methods considered time-dependent clearance and concentration–time points collected in earlier intervals. In conclusion, although AUC estimates between the two methods showed good correlation, in a simulated study, MAP lead to higher target attainment. When changing from one method to another, or changing infusion duration and other factors, optimum estimated exposure targets may require adjusting to maintain a consistent exposure.

**Supplementary Information:**

The online version contains supplementary material available at 10.1007/s10928-024-09915-w.

## Introduction

Methods of estimating area under the concentration–time curve (AUC) based on measured drug concentrations can be broadly grouped into non-compartmental analysis (NCA) and compartmental approaches. This latter group includes both regression methods of fitting an assumed pharmacokinetic (PK) model to the available measurements and Bayesian methods of balancing the assumed PK model priors with available measurements. NCA allows for estimation of certain exposure parameters and PK characteristics with fewer assumptions, but still assumes that elimination between samples in the elimination phase is first-order, that the half-life can be obtained by regression from the last few samples in the elimination phase, and that concentrations rise linearly during infusion (when using log-linear NCA) [1]. For drugs like busulfan with narrow therapeutic AUC ranges, dose adjustments based on AUC estimation have become a part of routine clinical practice [2] however dose adjustment recommendations for a given clinical scenario vary considerably from site to site due, in part, to differences in AUC estimation methods [3, 4]. With the growing availability of precision dosing software and other computational tools, model-based Bayesian methods are increasingly being adopted in clinical practice, although NCA remains a widely used standard. Because these two methods vary in the assumptions they make and in how they leverage patient data to inform their calculations, they vary in their estimations of AUC, and these differences could impact dose selection and patient outcomes.

**Fig. 1:**
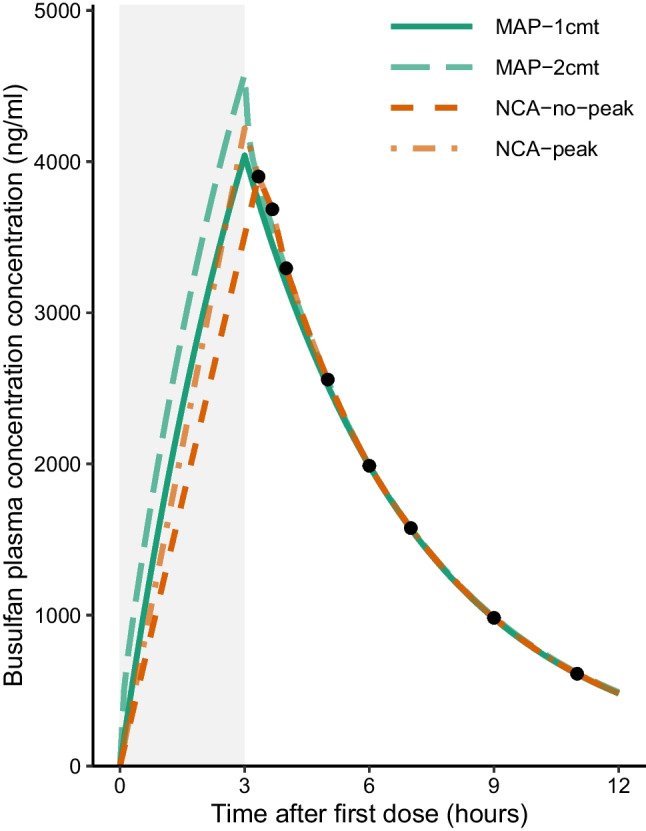
Comparison of two non-compartmental analysis (NCA) approaches with a compartmental model-based approach (MAP). The example patient was a fifteen-year-old male patient (weight: 72 kg, height: 183 cm) who received 229 mg of busulfan infused over 3 h as part of a once-daily 4-day regimen. Samples were collected at 20 min, 40 min, 1 h, 2 h, 3 h, 4 h, 6 h, 8 h after the end of infusion. AUC was estimated using non-compartmental analysis without peak extension or with peak extension, or using maximum a posteriori (MAP) Bayesian estimation using a one-compartment model (1cmt) [5] or a two-compartment model (2cmt) [6]. The curve has been restricted to the first 12 h for clarity

While NCA does not make explicit assumptions about how drugs distribute within and are cleared from the body, when implemented using the “log-linear” method it still assumes first-order decay for parts of the curve not described by samples. Consider the hypothetical patient in Fig. 1, who received a three-hour infusion of busulfan as part of a Q24 dosing regimen, and then provided eight serum samples, the first of which was collected 20 min after the end of infusion, and the last of which was 8 h after the last dose. AUC for the sampled regions of the curve is calculated using trapezoids, assuming logarithmic decay between samples. Typically, the AUC for the portion of the concentration–time curve after the final measurement is estimated by fitting a linear regression model on the log-transformed final three or four concentrations, then extrapolating to the time of the next dose (*C*_*Tau*_) or to infinity (to calculate cAUC after all doses are administered) [1]. The infusion portion of the curve is modeled linearly, rising from 0 (or some other pre-dose measurement) to the maximum concentration, *C*_*max*_. There are two ways to define *C*_*max*_: this value can either be the first measured concentration (Fig. 1: NCA-no-peak) or it can be estimated by extrapolating backwards from the first measured level to the end of infusion using the same regression principles for *C*_*Tau*_ (Fig. 1: NCA-peak). These two methods for estimating *C*_*max*_ will differ more the larger the time delay between the end of infusion and the first sample collection.

Model-based approaches, in contrast, assume some number of drug distribution compartments, and possibly other mathematical relationships describing distribution and clearance. Bayesian methods of updating model parameters to incorporate measured drug concentrations also assume distributions of inter-individual and residual variability. Maximum a posteriori (MAP) Bayesian estimation uses these distributions to identify point estimates of each PK parameter based on maximum likelihood, while full Bayesian approaches, which we do not further explore here, would additionally consider parameter uncertainty and use the full posterior for inference. Using these Bayesian PK parameter estimates, AUC is then estimated by integrating the concentration–time curve. As a result, the sampled region will be described by a smooth curve instead of trapezoids (Fig. 1: MAP). For a one-compartmental model, the shape of the curve and AUC estimated approximates the logarithmic decay of NCA in the part of the curve extrapolated to *C*_*tau*_. The two-compartment model shows some differences in concentration earlier during elimination. Differences between this PK model approach and the NCA methods are quite discernible in the infusion phase: model-based methods assume drug clearance during the infusion time, resulting in a non-linear curve. This discrepancy will be more pronounced the longer the infusion time.

In addition to assumptions about the shape of the concentration–time curve, these methods differ in what information is used to estimate the AUC for a given dose. NCA methods use only data collected within a single dosing interval, along with a pre-dose trough level. In contrast, Bayesian methods are informed by data from all prior dosing intervals, which may improve PK parameter estimation accuracy as additional data becomes available. Additionally, Bayesian methods incorporate some quantity of measurement error and other error in their likelihood estimates, providing some protection from background noise [7].

To better understand the practical implications of the differences between these methods, we compared differences in AUC estimated using NCA and Bayesian model-based estimation in a routine clinical care data set. Then, because “true” AUC cannot be measured without prohibitively densely sampled curves, we evaluate the ability of these methods to bring patients to a target AUC by simulating the clinical implementation of both approaches.

## Methods

### Data source

All patients or their guardians provided informed written consent to routine collection of therapeutic drug monitoring (TDM) of busulfan as part of pre-conditioning prior to stem cell transplantation. A waiver for informed consent to participation in this pharmacokinetic analysis was granted by the University of California, San Francisco (UCSF)’s Institutional Review Board because this retrospective review of de-identified data was assessed to involve no more than minimal risk to the subjects. Patients underwent autologous or allogeneic transplantation for a variety of malignant and nonmalignant disorders. All preparative regimens were busulfan-based, with a cumulative AUC goal ranging from 15-90 mg·h/L, depending on disease and combination of pre-transplant agents. For seizure prophylaxis, all subjects received levetiracetam beginning 24 h prior to the first dose of busulfan through 24 h post the last dose of busulfan. Moderate or potent enzyme inhibitors or inducers were not allowed during busulfan-based conditioning and discontinued prior to conditioning based on recommendations published by Winger et al. [8].

Patients with busulfan doses and TDM records were entered into the InsightRX Nova model-informed precision dosing software platform were included in this study. Patients were excluded from analysis if their dosing records could not be unambiguously interpreted or suggested unusual deviation from standard protocol, including the following reasons: multiple concurrent drug concentration–time points with discrepant values (*N* = 1), absence of samples collected within the first 150 min after the end of infusion (*N* = 1), doses administered less frequently than every 5 h (*N* = 1), or samples collected during infusion (*N* = 5).

### Clinical characteristics

Clinical characteristics of patients were calculated using the open-source R package clinPK [9]. Ideal body weight (IBW) was calculated according to the Devine equation for patients over the age of 18 years [10]. For patients under the age of 1 year, IBW was calculated as the patient’s weight. For patients between the age of 1 and 18 years with a height under 5 feet, IBW was calculated as 0.165 times the height in meters. For patients of this age range over 5 feet in stature, IBW was calculated as 39 kg (males) or 42.2 kg (females) plus 2.27 kg per inch over 5 feet. Fat-free mass was calculated using the Al-Sallami equation for patients under the age of 18 years [11] and the Janmahasatian equation [12] for patients over the age of 18 years. Fat mass was calculated as the difference between weight and fat-free mass.

### Pharmacokinetic modeling

NCA was performed using clinPK, with the option to extrapolate ${C}_{max}$ backwards to the end of infusion either set to true (“peak extension”) or false (“no peak extension”). Model-based PK parameter estimation was performed using two population pharmacokinetic models for busulfan (Supplementary Table 1): the McCune model is a two-compartment model developed on a data set of 1610 pediatric and adult patients [6] and the Shukla model is a one-compartment model developed on 199 pediatric and young adult patients that included some of the patients in the data set reported here [5]. The published McCune model reported inter-occasion variability (IOV) on clearance, both volumes of distribution and intercompartmental transfer, however our experience in using this model in clinical settings indicates that this level of flexibility leads to instability during MAP estimation. As a result, IOV was included only on clearance and the central volume of distribution. Model files are available open source at github.com/InsightRX/PKPDsim.

AUC was estimated iteratively for each dosing interval for which concentration–time points were available. For PK model approaches, maximum a posteriori (MAP) Bayesian estimation of the individual’s PK parameters was performed with the BFGS algorithm implemented in R’s stats::optim function [13], using the package PKPDsim for simulation of the concentrations and calculation of the likelihood [14]. These estimates were made using all samples collected up to and including that dosing interval in its posterior distributions, and these PK parameters were then used to estimate AUC by integrating the concentration–time curve.

### Simulated treatment

The impact of AUC estimation method on dose selection and cumulative AUC (cAUC) was evaluated using simulation. Simulated patients were given an initial dose of 3.2 mg/kg, and doses were adjusted to achieve a cAUC of 90 mg⋅h/L divided across four doses given every 24 h, infused over 3 h. After the first dose, new doses were calculated based on AUC estimates obtained either using NCA with peak extension or using MAP Bayesian estimation with the Shukla model. Concentration–time curves arising from selected dosing regimens were simulated using the McCune model, intentionally resulting in model misspecification between the simulation model and estimation model. Because the McCune model was used for simulation, we refer to AUC estimates extracted from these simulated curves as the true AUC within the analysis. Covariates for the simulated patients were mimicked from the actual patient data set, producing a realistic distribution of covariates. Pharmacokinetic parameters were created for the simulated patients by randomly sampling the interindividual variability and inter-occasion distributions described by the McCune model ten times, creating a data set of patients tenfold greater than the real data set. The same simulated patients were used for all experiments, for both NCA and MAP. TDM collection was simulated by extracting the concentration at standard collection times (15 min, 1 h, 3 h, 5 h after the end of infusion), and adding random noise, according to the proportional and additive residual error described by the McCune model. As before, IOV was included only for the central compartment parameters. True cAUC was calculated by integrating under the simulated concentration–time curve from the start of the first dose to 120 h after the final dose (at which point concentration approaches zero). Estimated cAUC was calculated using estimated PK parameters to simulate a concentration–time curve and then integrating under it from the first dose to 120 h after the final dose.

## Results

### Data collection

The final data set included 246 patients, who contributed 2455 busulfan concentration–time points from 568 busulfan administrations, summarized in Table 1. Patients were treated according to several conditioning regimens: busulfan/clofarabine/fludarabine (14% of patients), busulfan/fludarabine/cyclophosphamide, busulfan/fludarabine/thiotepa, or busulfan/melphalan. A more precise breakdown of conditioning regimens was not available in the data. Following the recent implementation of Bayesian forecasting software, standard practice at UCSF is to collect four samples per dosing interval (77% of dosing intervals); more densely sampled intervals were relatively rare (16% of intervals had six or more samples collected).

**Table 1:** Patient characteristics of the final data set

Characteristic	Median (range) or N	Inter-quartile range or %
Number of patients (% < 18 years)	246	69%
Number of TDM samples	2455	
Conditioning regimen:		
*Busulfan/Clofarabine/Fludarabine* *Other*	35 211	14% 86%
Age (years)	6.9 (0.2–65.7)	1.7–30.5
Sex		
*Female* *Male*	95 151	39% 61%
Total body weight (kg)	23 (5–155)	11–65
Height (cm)	118 (55–201)	81–165
Body mass index (kg/m^2^)	17 (12–55)	16–24
Body mass index (kg/m^2^) – adults only < 18.5 18.5–24.9 25–29.9 30–34.9 35–39.9 40 +	26 (17–55) 2 28 31 10 2 4	24–29 2.6% 36.4% 40.3% 13.0% 2.6% 5.2%
Weight category – children only		
< 9 kg 9–15.9 kg 16–22.9 kg 23–33.9 kg > 34 kg	49 45 30 19 26	29% 26.6% 17.8% 11.2% 15.4%
Ideal body weight (kg)	23 (4.9–94)	11–58
Fat-free mass (kg)	18 (4.0–80)	8.6–47
Fat mass (kg)	4.9 (0.8–91)	2.2–18

### AUC Estimation in real patients

**Fig. 2:**
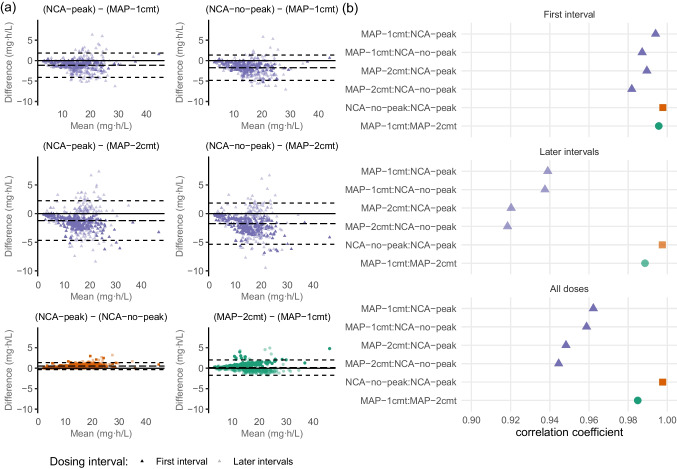
Agreement between AUC estimation methods. **a** Bland–Altman plots showing agreement between AUC estimation approaches. Each point representsan AUC estimate made by the corresponding method for a single dosing interval. Dashed lines indicate mean difference and the limits of agreement (mean difference ± 1.96 standard deviations). **b** Correlation coefficient describing correlation between AUC estimates for each dosing interval. NCA: non-compartmental analysis. MAP: maximum a posteriori estimation with either the one-compartment Shukla model (1cmt) or the two-compartment McCune (2cmt) methods on real patient data

The mean AUC estimated per dosing interval across all methods was 15.0 mg⋅h/L, and the four methods of AUC estimation showed good agreement in their estimation, as assessed by Bland–Altman plots (standard deviation of differences (SD) of 0.44–1.83 mg⋅h/L (i.e., 3–12% of mean AUC); Fig. 2a) and by correlation in estimates (correlation coefficients of 0.945–0.998; Fig. 2b). The two NCA methods showed the closest agreement (mean difference in AUC estimation of 0.53 mg⋅h/L, SD of 0.44 mg⋅h/L, and correlation coefficient of 0.998), which is to be expected since these methods differ only in how they estimate the shape of the concentration–time curve up until the time of the first sample collection. NCA with peak extension will always be higher than NCA without peak extension (see Fig. 1), explaining the bias observable in the Bland–Altman plot. The two PK models also showed close agreement (mean difference of 0.15 mg⋅h/L, SD of 0.96 mg⋅h/L, correlation coefficient of 0.985), suggesting that the two models arrive at similar PK parameter estimates despite differences in model structure and inter-individual variability. When comparing MAP estimates to NCA approaches, agreement was better for the first dosing interval (mean difference of 0.8–1.96, SD of 0.61–1.34, correlation of 0.982–0.994), but lower for subsequent dosing intervals (mean difference of 0.99–2.0, SD of 1.85–2.12, correlation of 0.918–0.938), with NCA methods consistently estimating lower exposures than MAP methods.

### Treatment simulations

**Fig. 3:**
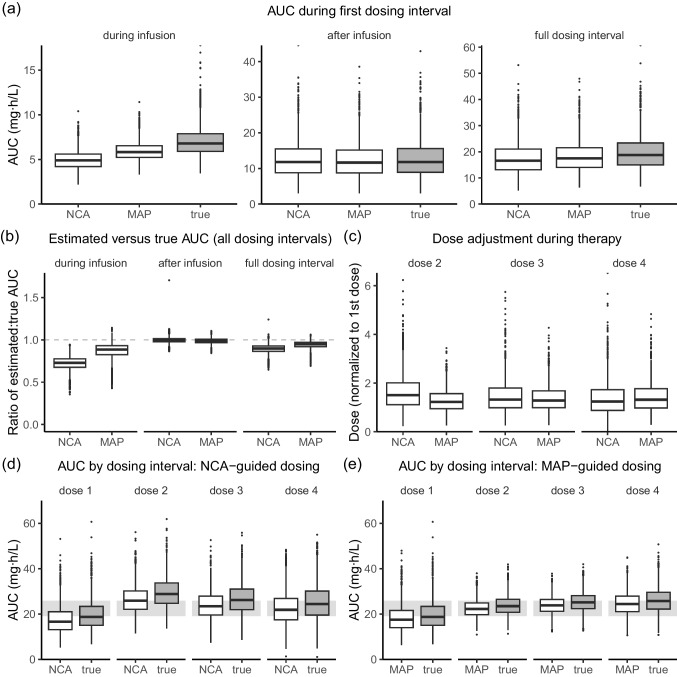
Differences between non-compartmental analysis (NCA) and maximum a posteriori Bayesian estimation (MAP) in AUC estimation and dose adjustment in a simulated trial. **a** AUC estimated following the first weight-based dose during infusion (t = 0–3 h), after infusion (t = 3–24 h), or across the full first dosing interval (t = 0–24 h). **b** Ratio of estimated AUC to true AUC across dosing intervals. **c** Doses selected by dosing interval, normalized to the initial dose to account for body-size dependence in doses. **d** AUC by dosing interval using NCA estimates for dose adjustment. **e** AUC by dosing interval using MAP for dose adjustment. Shaded grey region indicates a target AUC of 22.5 mg⋅h/L per day ± 15%. Concentration–time curves were simulated with the McCune model (true AUC) and estimated with NCA or with MAP using the Shukla model. Samples were collected at 3.25, 4, 6, and 8 h after the start of infusion. Boxplot indicates the median (bold line), 2^5^th-7^5^th percentiles (outer rectangle) and 1.5 × the interquartile range from the 2^5^th-7^5^th percentiles (whiskers)

In the simulated treatment courses, since initial dosing was weight-based, the first dose was the same for both the NCA and MAP arms. AUC estimates arising from the first dose across the full dosing interval varied by estimation method (Fig. 3a). The mean AUC estimated by NCA was 17.6 mg⋅h /L while the mean AUC estimated by MAP was 18.3 mg⋅h/L, compared to a true mean AUC of 19.7 mg⋅h /L. However, the majority of the variation arose from differences in AUC estimation during infusion: the mean AUC during infusion was 4.97 mg⋅h /L for NCA and 5.93 mg⋅h /L for MAP compared to 7.02 mg⋅h/L true AUC. In contrast, AUC estimates after infusion were similar (NCA: 12.6 mg⋅h/L, MAP: 12.3 mg⋅h/L, true: 12.7 mg⋅h/L). This trend was true for later dosing intervals too, with NCA underestimating AUC by 28% during infusion and 0.4% after infusion, and MAP underestimating AUC by 13% during infusion and 1.31% after infusion (Fig. 3b). (The differences in estimated and true AUC have been expressed as proportions and not in absolute AUC values as they cannot be compared between methods after dose adaptation has been performed.) That the two-compartment model (designated the true AUC in this experimental design) produces a higher AUC than the one-compartment model and the NCA method is consistent with the sharp peak visible in Fig. 1.

Following the first dose, subsequent doses were adjusted based on AUC estimates to attain a cAUC of 90 mg⋅h/L (Fig. 3c, expressed normalized to the initial dose to control for differences in patient weight). The weight-based starting dose of 3.2 mg/kg per day, which is more aggressive than the FDA label recommendation of 3.2 mg per kilogram adjusted body weight (the minimum of IBW and total body weight) divided over four doses, led to underexposure during this first dosing interval (Fig. 3d, e). Both estimation methods correctly identified this underexposure and increased dosing accordingly. However, the value of the second dose greatly varied between the two methods (Fig. 3c): the second dose was on average 63% higher than the first dose for NCA, and 28% higher than the first dose for MAP (paired t-test comparing NCA versus MAP: *p*-value < 0.0001). In part, this difference is related to how the PK models describe clearance over time. Busulfan clearance is reported to decrease over time [5, 6, 15], and both PK models incorporate this change in clearance. In the McCune model, clearance decreases by 6.8% after 6 h of therapy and by 8.1% after 36 h of therapy relative to initial clearance, while in the Shukla model, clearance decreases by 13.5% after 24 h of therapy. In contrast, NCA does not incorporate time-dependent changes in clearance. As a result, this method overpredicted busulfan clearance on day 2 of therapy, resulting in too high of a dose and too high an exposure (Fig. 3c–d). To balance out this overexposure, doses and exposures for day 3 and day 4 were, on average, lower. Because the Shukla model expects a larger decrease in clearance from day 1 to day 2 than described in the model used for simulation (McCune), it resulted in a smaller dose than necessary to make up the underexposure from day 1, and as a result, doses and exposures were higher for day 3 and day 4 (Fig. 3c,e).

The variation in dose recommendations and estimated AUC was substantially narrower in MAP-guided dose adjustment, consistent with prior prospective trials [5]. MAP-guided dose adjustment also led to less variation in true AUC (Fig. 3d–e).

**Fig. 4:**
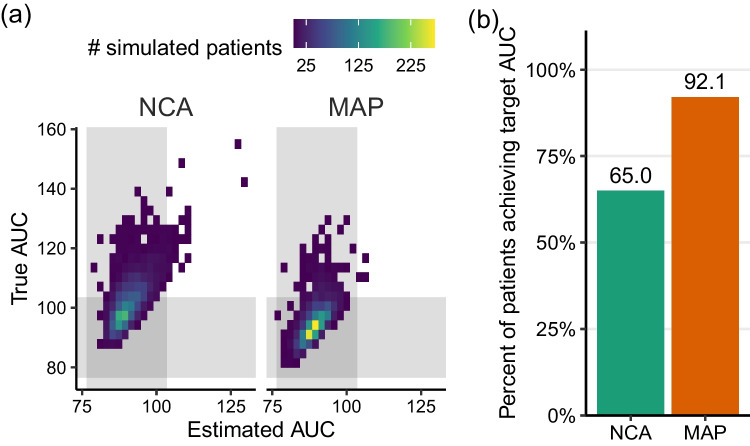
Cumulative AUC target attainment in a simulation study using either non-compartmental analysis (NCA) or maximum a posteriori Bayesian estimation (MAP) to estimate AUC and personalize doses. **a** True AUC versus estimated AUC by method, with shaded regions indicating the target AUC (90 mg⋅h/L) ± 15%. **b** Percentage of simulated patients achieving a true cumulative AUC within 15% of the target

Attainment of the cAUC target also varied by estimation method. Simulated patients were deemed “on target” if their cumulative exposure was within 15% of the target cAUC of 90 mg⋅h /L. For both estimation methods, estimated target attainment following all four doses was high: 98.7% for NCA and 99.9% for MAP (Fig. 4 and Table 2, “4 samples”). However, both methods overestimated target attainment compared to true target attainment. This misestimation was considerably larger for NCA (65.0% true target attainment) than for MAP (92.1% true target attainment). Variability in estimated cAUC was higher for simulated patients dosed using NCA (standard deviation: 4.5 mg⋅h/L) compared to MAP (standard deviation: 3.3 mg⋅h/L), consistent with findings in real patients [5]. True variability was higher than estimated variability in both methods (standard deviation, NCA: 7.6 mg⋅h/L; MAP: 5.7 mg⋅h/L).

**Table 2:** Summary statistics of true and estimated area under the concentration–time curve (AUC) by estimation method and sampling times. Values are in mg·h/L unless otherwise indicated

Sampling	Method	Estimation error		Percent on target		Median (range)		Mean (SD)	
		nRMSE	MPE	Estimated	True	Estimated	True	Estimated	True
3 samples t = 3.25, 4, 6 h	NCA	12.9%	−11.1%	98.6%	66.0%	90 (79–126)	101 (84–153)	91 (4.5)	102 (8.2)
	MAP	8.4%	−6.3%	99.9%	92.2%	89 (77–104)	95 (81–135)	89 (3.2)	96 (6.1)
3 samples t = 3.25, 5, 8 h	NCA	12.2%	−10.7%	98.5%	62.6%	90 (79–127)	100 (86–155)	91 (4.5)	102 (7.6)
	MAP	7.8%	−6.1%	100%	90.8%	89 (77–106)	95 (81–127)	89 (3.3)	95 (5.6)
4 samples t = 3.25, 4, 6, 8 h	NCA	12.3%	−10.7%	98.7%	65.0%	90 (79–129)	101 (87–156)	91 (4.5)	102 (7.6)
	MAP	7.9%	−6.1%	99.9%	92.1%	89 (76–107)	94 (81–130)	89 (3.3)	95 (5.7)
5 samples t = 3.25, 4.5, 6, 8, 11 h	NCA	12.0%	−10.5%	98.7%	66.2%	90 (79–127)	100 (87–157)	91 (4.5)	102 (7.4)
	MAP	7.6%	−5.9%	99.8%	93.0%	89 (76–107)	94 (78–128)	89 (3.3)	95 (5.6)
dense, full interval	NCA	3.6%	−3.1%	98.5%	67.6%	90 (82–119)	93 (84–127)	91 (3.9)	94 (4.4)
	MAP	5.1%	3.1%	99.8%	91.3%	89 (78–105)	87 (62–107)	89 (3.1)	87 (4.8)
dense, post-infusion only	NCA	11.8%	−10.3%	99.3%	97.3%	90 (79–128)	100 (87–156)	91 (4.4)	101 (7.4)
	MAP	8.1%	−6.2%	99.9%	96.6%	89 (77–107)	94 (82–138)	89 (3.4)	96 (6.0)

*nRMSE* normalized root mean square error, *MPE* mean percent error, *SD* standard deviation, *NCA* non-compartmental analysis, *MAP* maximum a posteriori Bayesian estimation

This simulated trial used the sampling strategy that is standard practice at UCSF. Increasing the number of samples to five samples per interval (collected at sample times described by Yeh et al. [16]) to include a final sample at 11 h improved true target attainment slightly for both estimation methods (Supplementary Fig. 1 and Table 2, “5 samples”. NCA: 66.2%, MAP: 93%). Reducing the samples per interval to three did not adversely impact true AUC estimation when the final sample at 8 h was retained but did lead to lower true target attainment when the final sample was at 6 h (Supplementary Fig. 1 and Table 2). Because the McCune model has two compartments, this later time point is likely quite informative for estimating clearance during the elimination phase. This model also includes mid-interval changes in clearance (at 6 and 36 h after the first dose), and so later collection points (samples at 8 h, 11 h) would also allow better capture of these dynamics.

Interestingly, increasing sample density to every 30 min starting 15 min after the end of infusion did not substantially improve true target attainment (Supplementary Fig. 1 and Table 2, “dense, post-infusion”: NCA: 67.6%, MAP: 91.3%). Hypothesizing that this difference between NCA and model-based AUC may be due to mis-estimations of AUC during infusion (Fig. 3a–b), we repeated this experiment with samples during infusion and observed a marked increase in target attainment for both estimation strategies (Supplementary Fig. 1 and Table 2, “dense, full interval”: NCA: 97.3%, MAP 96.6%).

In the real-world data, NCA and MAP showed greater disagreement after the first dosing interval (Fig. 2). Hypothesizing that this difference was because NCA uses samples only from the most recent dosing interval while MAP can leverage all samples collected to-date, we lastly assessed the impact of using MAP with only samples from the most recent dosing interval (Supplementary Fig. 2). Overall, the difference in error in estimating true AUC was relatively minor, dropping to a mean absolute percent error of 6.1% when only samples from the most recent interval were used compared to 5.8% in standard MAP. This difference in estimation error resulted in a decrease in true target attainment to 90.3%, a reduction of 1.8 percentage points.

## Discussion

With busulfan as a case study, we examined how algorithmic differences between NCA and MAP impact AUC estimation and target attainment, using a combination of real-world data and simulated trials. We identified three key sources of differences: how concentration–time dynamics during infusion are modeled, how time-dependent clearance is modeled, and which samples are considered during estimation of AUC after the first dosing interval.

NCA models infusion linearly, assuming elimination during this phase is negligible. PK model-based approaches include elimination during infusion, although differences in the shape of this phase arise due to assumptions about model structure (i.e., one-compartment, multi-compartment). In the simulated trial, both NCA and MAP estimated true AUC between the end of infusion and the end of the dosing interval accurately and with minimal bias. However, there were substantial differences in estimation of AUC during the infusion phase. These differences translated to sizable differences in AUC estimates across the overall dosing interval, and impacted dose adjustment and true simulated AUC target attainment. When samples were collected during infusion, target attainment improved substantially for both methods.

Simulations of sample collection during infusion are likely over-optimistic: residual variability is likely higher during infusion due to factors that most popPK models do not account for, such as incomplete distribution or non-linear drug delivery. Our simulations assumed doses were fully administered at a consistent rate; clinical considerations like method of infusion can lead to non-linearity in drug delivery [17], which would increase error in estimation of true AUC for both estimation methods. Still, our results indicate AUC during infusion as an important source of variability between estimation methods. For drugs dosed over relatively long time periods, the AUC during infusion can make up a significant portion of the total AUC, so this error can be quite clinically meaningful; in our study, AUC during the 3-h infusion comprised about a third of the total AUC. For drugs dosed by bolus or over shorter infusions, the two estimation methods would likely show better agreement. Despite our study’s relatively long infusion period, the two methods showed strong agreement on real data (R^2^ of 0.982–0.994) during the first dosing interval, in which the same number of samples are used to inform AUC estimation.

Historically, busulfan was given four times per day, infused over two hours (one third of the dosing interval). Shifting to a proportionally larger dose administered once per day over three hours (one eighth of the dosing interval) was considered more convenient for patients and staff and did not result in statistically different daily AUC, clearance or clinical outcomes [18, 19]. However, these studies were relatively small; it’s possible that there were indeed differences in error in estimation of true AUC that could lead to differences in patient outcomes. Interestingly, the once-daily dose, with less of the dosing interval spent during infusion, may correspond to greater agreement between NCA and MAP. However, when changing from one estimation method to another, clinicians should be cautious about assuming AUC targets remain the same for the reasons we’ve shown here. In our own experience, the shift from NCA to MAP coincided with a change in AUC target from 72 to 90 mg⋅h/L, likely partially due to the discrepancies in AUC estimation described here [20]. This tendency of NCA to underestimate AUC relative to MAP has previously been reported [4].

Clearance of busulfan is widely reported to decrease over therapy [5, 6, 20]. The mechanism underlying this decrease is not well-known, however may arise due to glutathione depletion [15, 21]. NCA does not consider time-dependent changes in patient PK, and as a result, tended to overdose patients on day 2 of our simulated trial. PopPK-based methods can account for this decrease in clearance over time, potentially leading to more appropriate dose selection [22]. The models used for simulation and estimation differed in how they modeled this time-dependent clearance, resulting in slight *under-*dosing on day 2 in the MAP arm of our simulated trial, and pinpointing a need for accurate description of time-dependent changes in clearance. The true physiological picture is likely more complicated than considered in our simulated trial: glutathione levels vary between individuals and decline with age [15], which neither of the two popPK models used here consider, and other metabolic pathways may be involved as well [23]. Additionally, both models use step functions to describe continuous physiological processes.

One benefit of MAP is that samples from the full treatment course inform PK parameter estimates. For example, for selection of the fourth dose, MAP considers drug concentration–time points collected across three prior dosing intervals, while NCA considers only concentration–time points from the third dosing interval. When MAP estimation was restricted to samples from the most recent dosing interval, AUC estimation became slightly more erroneous, and led to a slight decrease in target attainment. MAP also revises past estimates as new data becomes available, while NCA estimates are “set in stone”, which may improve estimation accuracy.

Of these three sources of variation in AUC estimation and target attainment between NCA and MAP, differences in description of the infusion phase played the largest role. We also considered the impact of sample timing on AUC target attainment. This effect can be more intuitively understood knowing about these other sources of variability: a wider spread in sample collection times better captures time-dependent changes in clearance, while samples collected during infusion help inform estimates of AUC during infusion. Because of algorithmic differences, optimal sampling times likely vary between methods of estimation, although a full analysis of optimal sampling is outside the scope of this work.

There are some estimation methods we did not consider here. For example, MAP estimation with a reduced (“flattened”) prior or least-squares estimation (essentially setting the weight of the prior to zero) could be used [7]. Both these methods listen more closely to the data — like NCA — but like MAP, can incorporate covariate effects like time-dependent changes in clearance and leverage samples collected in all dosing intervals. We would therefore expect these estimation methods to behave more similarly to MAP, perhaps with more sensitivity to measurement error.

Although our work finds that MAP leads to higher AUC target attainment and reduced variability in doses in a simulated precision dosing trial, NCA will remain an important analytical tool. MAP requires a sufficiently fit-for-purpose popPK model, which is not always available. NCA, due to its lack of compartmental assumptions, will remain the gold standard for estimation of drug exposure and regulatory reporting in early-phase drug development. NCA could also be helpful for precision dosing of patients for whom no popPK model is suitable. Some under-served patient populations are typically excluded from model development, such as amputees and pregnant patients [24], and so having both estimation methods available may improve the equity and inclusivity of precision dosing.

This work used simulation with intentional model misspecification between the model used for simulation and estimation to assess the ability of these methods to determine true AUC, which cannot be empirically measured. It should be emphasized that both NCA and MAP *estimate* AUC; neither method can be considered “perfect”. However, our work here shows that due to underlying methodological differences and mathematical assumptions, MAP Bayesian estimation outperforms NCA for dose personalization for busulfan dosing, and these findings will likely translate to other MIPD applications.

### Supplementary Information

Below is the link to the electronic supplementary material.

Supplementary file1 (DOCX 69 KB)

Supplementary file2 (DOCX 428 KB)

Supplementary file3 (XLSX 8817 KB)

Supplementary file4 (DOCX 18 KB)

Supplementary file5 (CSV 166876 KB)
