# Supplementary material for: Maximum a posteriori Bayesian methods out-perform non-compartmental analysis for busulfan precision dosing
Source: J Pharmacokinet Pharmacodyn. 2024 Mar 23;51(3):279–88. doi: 10.1007/s10928-024-09915-w (PMC11136738; doi:10.1007/s10928-024-09915-w)
Supplement: Supplementary file 1 — Supplementary file1 (DOCX 69 KB) [file 10928_2024_9915_MOESM1_ESM.docx]

**Supplementary Figure 1:** True versus estimated target attainment based on sampling times. Simulated concentration-time points were collected at the specified times following each dose, with dense sampling indicating samples every 30 minutes starting at t = 3.25 h (post-infusion only) or t = 0.25 h (full interval) and ending at t = 23.25 h after each dose. Shaded grey regions indicate the target AUC (90 mg⋅h/L) ± 15%.
