# Supplementary material for: Maximum a posteriori Bayesian methods out-perform non-compartmental analysis for busulfan precision dosing
Source: J Pharmacokinet Pharmacodyn. 2024 Mar 23;51(3):279–88. doi: 10.1007/s10928-024-09915-w (PMC11136738; doi:10.1007/s10928-024-09915-w)
Supplement: Supplementary file 2 — Supplementary file2 (DOCX 428 KB) [file 10928_2024_9915_MOESM2_ESM.docx]

** Supplementary Figure 2**: Effect of using only samples from the most recent dosing interval on MAP AUC estimation. (a) Correlation between MAP using all concentration-time points and MAP using concentration-time points from the most recent interval only. The first dosing interval was excluded for clarity since these fall along the line of identity. (b) Decrease in mean absolute percent error (MAPE) in estimating true AUC when using samples from the most recent interval only or all dosing intervals to-date. (c) AUC (mg⋅h/L) in a simulated trial when dose adjustment is performed using MAP with concentration-time points only from the most recent dosing interval (see also Supplementary Figure 1, “4 samples”). Shaded grey regions indicate the target AUC (90 mg⋅h/L) ± 15%.
