# Supplementary material for: Maximum a posteriori Bayesian methods out-perform non-compartmental analysis for busulfan precision dosing
Source: J Pharmacokinet Pharmacodyn. 2024 Mar 23;51(3):279–88. doi: 10.1007/s10928-024-09915-w (PMC11136738; doi:10.1007/s10928-024-09915-w)
Supplement: Supplementary file 4 — Supplementary file4 (DOCX 18 KB) [file 10928_2024_9915_MOESM4_ESM.docx]

**Supplementary Table 1:** Comparison of the McCune model and the Shukla model

| **Characteristic** | | **Unit** | **McCune** | **Shukla** |
| --- | --- | --- | --- | --- |
| *Development information* | | | | |
|  | # Patients |  | 1610 | 199 |
|  | # Concentration time points |  | 12,380 | 3580 |
|  | Patient population |  | Pediatric + adult, HCT | Pediatric, HCT |
| *Pharmacokinetic parameters* | | | | |
|  | CL | L/h | $11.4\cdot\left( FFM+0.509\cdot FM \right)\cdot FMAT\cdot\theta_{t}$ | $3.96\cdot F_{mat}\cdot\left( \frac{FFM}{12} \right)^{0.75}\cdot{0.8}^{REG}\cdot\theta_{t}$ |
|  | V | L | $13.9\cdot\left( FFM+0.203\cdot FM \right)\cdot{1.07}^{SEX}$ | $10.8\cdot\frac{FFM}{12}$ |
|  | V2 | L | $29.9\cdot\left( FFM+0.203\cdot FM \right)\cdot{1.07}^{SEX}$ |  |
|  | Q | L/h | 135.2 |  |
|  | $\theta_{t}$ | % | $t\leq6:100$  $6<t\leq36:93.2$  $t>36:91.9$ | $t\leq24:100$  $t>24:86.5$ |
|  | $F_{mat}$ |  | $\left( 1+ \left( \frac{PMA}{45.7} \right)^{2.3} \right)^{-1}$ | $0.451+0.549\cdot\left( 1-e^{-1.37\cdot Age} \right)$ |
| *Interindividual variability* | | | | |
|  | CL | % | 21.5 | 24 |
|  | V | % | 41 | 17 |
|  | V2 | % | 12 |  |
|  | Q | % | 92.2 |  |
| *Inter-occasion variability* | | | | |
|  | CL |  | 11.3 | 12.9 |
|  | V |  | 22.4 | 13.3 |
| *Residual Error* | | | | |
|  | proportional | % | 3.87 | 10.6 |
|  | additive | ng/mL | 27 | 22.2 |

- FFM: fat-free mass.
  - Shukla model: calculated according to Al-Sallami et al. [11]
  - McCune model: calculated according to Janmahasatian [12]
- FM: Fat mass. $FM=Weight-FFM$
- REG: Regimen, where 1 = conditioning with clofarabine/fludarabine/busulfan, 0 = other.
- SEX: Patient sex, where female = 1.
- PMA: post-menstrual age, in weeks.
- HCT: Hematopoietic cell transplantation.
